# Repriming by PrimPol is critical for DNA replication restart downstream of lesions and chain-terminating nucleosides

**DOI:** 10.1080/15384101.2016.1191711

**Published:** 2016-05-26

**Authors:** Kaori Kobayashi, Thomas A. Guilliam, Masataka Tsuda, Junpei Yamamoto, Laura J. Bailey, Shigenori Iwai, Shunichi Takeda, Aidan J. Doherty, Kouji Hirota

**Affiliations:** a Department of Chemistry, Graduate School of Science and Engineering, Tokyo Metropolitan University, Hachioji-shi, Tokyo, Japan; b Genome Damage and Stability Center, School of Life Sciences, University of Sussex, Brighton, UK; c Department of Radiation Genetics, Graduate School of Medicine, Kyoto University, Yoshidakonoe, Sakyo-ku, Kyoto, Japan; d Division of Chemistry, Graduate School of Engineering Science, Osaka University, Toyonaka, Osaka, Japan

**Keywords:** chain-terminating nucleoside analogs (CTNAs), DT40, primase; primpol; polymerase; replication; repriming; restart

## Abstract

PrimPol is a DNA damage tolerance enzyme possessing both translesion synthesis (TLS) and primase activities. To uncover its potential role in TLS-mediated IgV_λ_ hypermutation and define its interplay with other TLS polymerases, *PrimPol*^−/−^ and *PrimPol*^−/−^*/Pol*η^−/−^*/Pol*ζ ^−/−^ gene knockouts were generated in avian cells. Loss of PrimPol had no significant impact on the rate of hypermutation or the mutation spectrum of IgV_λ_. However, *PrimPol*^−/−^ cells were sensitive to methylmethane sulfonate, suggesting that it may bypass abasic sites at the IgV_λ_ segment by repriming DNA synthesis downstream of these sites. *PrimPol*^−/−^ cells were also sensitive to cisplatin and hydroxyurea, indicating that it assists in maintaining / restarting replication at a variety of lesions. To accurately measure the relative contribution of the TLS and primase activities, we examined DNA damage sensitivity in *PrimPol*^−/−^ cells complemented with polymerase or primase-deficient PrimPol. Polymerase-defective, but not primase-deficient, PrimPol suppresses the hypersensitivity of *PrimPol*^−/−^ cells. This indicates that its primase, rather than TLS activity, is pivotal for DNA damage tolerance. Loss of TLS polymerases, Polη and Polζ has an additive effect on the sensitivity of *PrimPol*^−/−^ cells. Moreover, we found that PrimPol and Polη-Polζ redundantly prevented cell death and facilitated unperturbed cell cycle progression. *PrimPol*^−/−^ cells also exhibited increased sensitivity to a wide variety of chain-terminating nucleoside analogs (CTNAs). PrimPol could perform close-coupled repriming downstream of CTNAs and oxidative damage *in vitro*. Together, these results indicate that PrimPol's repriming activity plays a central role in reinitiating replication downstream from CTNAs and other specific DNA lesions.

## Introduction

Genome replication is stalled by DNA secondary structures and unrepaired damage, potentially leading to fork collapse, mutagenesis, and genome instability.^1^ Eukaryotic cells have evolved several mechanisms to complete replication beyond a damaged template. The first is translesion DNA synthesis (TLS), which employs specialized DNA polymerases, including Polymerase η and Polymerase ζ, to permit continued replication beyond the damaged template.^2-5^ A second mechanism is homologous recombination (HR), which mediates continuous replication using a newly synthesized sister strand.^6-8^ A third mechanism, in which DNA primases play a role, involves the repriming of DNA synthesis downstream from the lesion or structure.^9-13^

Primase-Polymerase (PrimPol) is a member of the archaeo-eukaryotic primase (AEP) superfamily,^14^ which was recently identified as a novel primase-polymerase that possesses both TLS polymerase and primase activities that are involved in DNA damage tolerance in eukaryotic organisms.^15-18^ PrimPol has two functional domains: an archaeo-eukaryotic primase (AEP) polymerase domain and a DNA-binding, zinc-finger domain.^13^ The AEP polymerase domain is required for both catalytic activities, while the zinc-finger domain is required only for primase activity,^13^ with a potential additional role in modulating polymerase fidelity and processivity to limit misincorporation during DNA synthesis. PrimPol's capacity to reprime appears to be important for maintaining replication fork progression at sites of UV damage^12,13^ and at structured DNA, such as G quadruplexes.^19^ Deletion of PrimPol in avian DT40 cells leads to replication fork slowing, damage sensitivity and a pronounced G2/M checkpoint arrest after UV irradiation.^20^ Deletion of a PrimPol ortholog PPL2 in trypanosomes is lethal.^17^ PrimPol is a very unprocessive enzyme with low fidelity and a mutation spectrum that is highly biased toward insertion-deletion errors.^21^ The activity of PrimPol appears to be modulated by its binding to single-stranded binding proteins.

A point mutation (tyrosine 89 to aspartic acid) located in the AEP polymerase domain of PrimPol has been identified in familial high myopia.^22^ The PrimPol^Y89D^ mutant enzyme is primase-active, but exhibits significantly reduced DNA polymerase activity.^23^ Mutation or deletion of the zinc-finger domain (PrimPol^ZF-KO^ and PrimPol^1-354^, respectively) in PrimPol leads to loss of primase activity but does not diminish primer extension synthesis, indicating that the zinc-finger domain plays a key role in the primase activity.^13^

Recent studies have shown that PrimPol's enzymatic properties, including its ability to initiate new DNA synthesis beyond several types of replicase stalling lesions and structures, help to maintain active replication.^12,15,16^ One current model of cellular TLS postulates that after replicative polymerases have halted at lesions, the stalled forks are restarted by specialized DNA polymerases, including Polη and Polζ, which are capable of TLS.^4,24^ The active sites of these enzymes are less spatially constrained than replicative polymerases and can thus accommodate distorted base-pairing involving damaged bases.^25,26^ Eukaryotic cells have evolved a number of TLS polymerases with distinctive bypass activities, though the division of labor among these various enzymes has not yet been fully elucidated. Likewise, although it is believed that PrimPol contributes to the cellular tolerance of DNA damage by facilitating damage bypass, the relationship between PrimPol and other TLS polymerases remains to be defined.

To better define the roles played by PrimPol in damage tolerance, we generated *PrimPol*^−/−^ and *PrimPol*^−/−^*/Pol*η^−/−^*/Pol*ζ ^−/−^ cells derived from the chicken DT40 B cell line. DT40 cells undergo TLS mediated IgV_λ_ hypermutation during *in vitro* passage. Thus, the unique advantage of this cell line for the phenotypic analysis of TLS is that the DNA sequence analysis of IgV_λ_ allows identification of nucleotides inserted opposite abasic sites by TLS. We show that the loss of PrimPol has no significant impact on the rate of hypermutation and mutation spectrum of IgV_λ_. However, *PrimPol*^−/−^ cells were sensitive to methylmethane sulfonate (MMS), which generates abasic sites. These data suggest that PrimPol may bypass abasic sites at the IgV_λ_ segment by repriming DNA synthesis downstream of the abasic sites. Consistently, the expression of a polymerase-deficient PrimPol^Y89D^ in *PrimPol*^−/−^ cells significantly suppressed this hypersensitivity, while the expression of primase-deficient PrimPol exhibited little suppression. Notably, *PrimPol*^−/−^*/Pol*η^−/−^*/Pol*ζ ^−/−^ cells proliferated very slowly and exhibited increased cell death, although both *PrimPol* ^−/−^ and *Pol*η^−/−^*/Pol*ζ ^−/−^ cells were able to proliferate with nearly normal kinetics. These results suggest that repriming by PrimPol and TLS by Polη and Polζ are compensatory for each other for facilitating physiological levels of DNA replication. Additionally, we found strong sensitivity of *PrimPol*^−/−^ cells to chain-terminating nucleoside analogs (CTNAs). Finally, we demonstrate that PrimPol is able to perform close-coupled repriming downstream of CTNAs and DNA damage lesions. Taken together, these data suggest novel roles for the primase activity of PrimPol in repriming DNA synthesis downstream of CTNAs and lesions incorporated on the primer strand that block 3′ extension by the replicative polymerases.

## Results

### DT40 cells lacking PrimPol are viable

In order to study the possible roles of PrimPol in TLS-dependent IgV hypermutation, we disrupted the *PrimPol* gene^15^ in Cl18, a subclone of DT40.^27^ This subclone has been used for analyzing HR-mediated gene conversion and TLS-dependent hypermutation events in immunoglobulin V_λ (_IgV_λ_) segments, because this cell line has a uniform IgV_λ_ sequence and change of sequence can be easily assessed by sequencing analysis.^2,3,5^ To disrupt the chicken *PrimPol* locus, we constructed two targeting vectors, *PrimPol-bsr* and *PrimPol-pur* (Fig. S1A) and sequentially transfected these constructs into *wild-type* DT40 cells. Targeted disruption on the *PrimPol* gene was verified by Southern blot analysis of *Eco*T22I-digested genomic DNA with the use of an internal 5′ probe (Fig. S1B) and RT-PCR (Fig. S1C). The *PrimPol* ^−/−^ cells proliferated with normal kinetics (Fig. S1D) but showed a slightly increased G2 population as previously reported (Fig. S1E).^15,20^

### PrimPol^−/−^ cells are hypersensitive to UV, MMS and cisplatin

**Figure 1.**
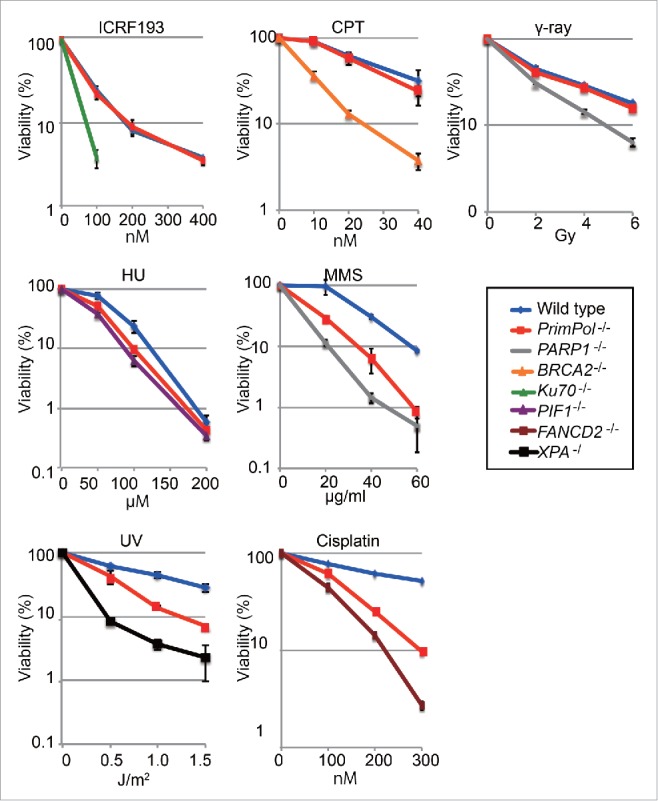
PrimPol-deficient cells are sensitive to a wide range of DNA damaging agents. *PrimPol* ^−/−^ cells exhibit hypersensitivity to various types of DNA damage. Cells with the indicated genotype were exposed to the indicated genotoxic agents. The dose of the genotoxic agent is displayed on the x-axis on a linear scale, while the percent fraction of surviving cells is displayed on the y-axis on a logarithmic scale. Error bars show the SD of the mean for 3 independent assays.

Previously, we reported that *PrimPol*^−/−^ cells exhibit UV, but not ionizing radiation sensitivity.^15,20^ To understand roles played by PrimPol more comprehensively, we measured sensitivity to a wider range of exogenous DNA damaging agents (cisplatin, HU, MMS, ICRF193 and camptothecin). *PrimPol*^−/−^ cells were not sensitive to ICRF193, camptothecin or γ-rays, confirming that PrimPol is not vital for strand break repair.^15,20^ Survival assays revealed that *PrimPol*^−/−^ cells were more sensitive to ultraviolet (UV), methylmethane sulfonate (MMS), cisplatin and hydroxyurea (HU) treatments than *wild-type* cells (Fig. 1). These data suggest that PrimPol plays roles in the restart of replication at sites of DNA damage. Hypersensitivity to a wide range of DNA replication blocking agents is also observed in *RAD18*^−/−^, *Pol*ζ^−/−^ and *PCNA* ^*K164R /K164R*^ cells,^2,28.29^ suggesting that lesion bypass is significantly impaired in *PrimPol*^−/−^ cells.

### PrimPol plays roles in damage tolerance independently of Polη and Polζ

**Figure 2.**
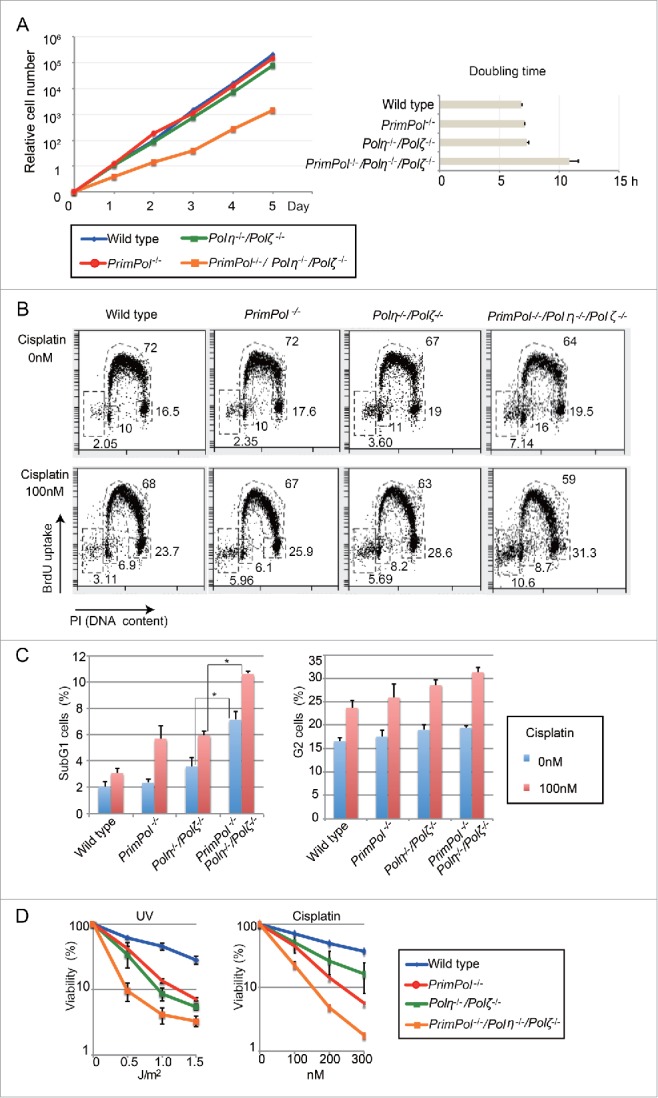
PrimPol plays roles in damage tolerance independently of Polη and Polζ. (A) Relative growth rate of cells plotted with indicated genotypes. Doubling time for the indicated cells was calculated. Error bars represent standard deviation from independent experiments (n = 3). (B) Indicated cells were treated with 0 or 100 nM of cisplatin for 16 hr. Representative cell-cycle distribution for the indicated genotypes. The top of the box, and the lower left, lower right, and left-most gates correspond to cells in the S, G_1_, and G_2_/M phases, and the sub-G_1_ fraction, respectively. The sub-G_1_ fraction represents dying and dead cells. The percentage of cells in each gate is indicated. (C) Percentage of the indicated cells in sub-G1 fraction and G2 phase fraction was indicated. Error bar represent standard deviation from independent experiments (n = 3). Statistical significance was determined by a Student's *t*-test and *p*-value was calculated. (*) *p* < 0.05 (D) Indicated cells were exposed to UV or cisplatin and sensitivities were indicated as in Figure 1.

Notably, we found that *PrimPol*^−/−^*/Pol*η^−/−^*/Pol*ζ^−/−^ cells proliferated more slowly than *wild-type, PrimPol*^−/−^ or *Pol*η^−/−^*/Pol*ζ^−/−^ cells (Fig. 2A). Moreover, *PrimPol*^−/−^*/Pol*η^−/−^*/Pol*ζ^−/−^ cells exhibited significantly higher levels of spontaneous cell death than observed with *wild-type, PrimPol*^−/−^^20^ or *Pol*η^−/−^*/Pol*ζ^−/−^ cells as evidenced by the observations that sub-G1 (dead cells) fraction in the cell cycle distribution was increased in *PrimPol*^−/−^*/Pol*η^−/−^*/Pol*ζ^−/−^ cells (Fig. 2B-C). In addition, *PrimPol*^−/−^*/Pol*η^−/−^*/Pol*ζ^−/−^ cells tended to exhibit more prominent G2 arrest after exposure to cisplatin than *wild-type, PrimPol*^−/−^ or *Pol*η^−/−^*/Pol*ζ^−/−^ cells (Fig. 2B-C). These results suggest that the repair kinetics of the DNA damage is critically reduced in *PrimPol*^−/−^*/Pol*η^−/−^*/Pol*ζ^−/−^ cells. Furthermore, the loss of Polη and Polζ increased sensitivity to DNA damage in *PrimPol*^−/−^ cells to the same extent as in the *wild-type* cells and, critically, the triple mutant was much more sensitive (Fig. 2D). These observations indicate that PrimPol and Polη-Polζ-dependent TLS contribute to DNA damage tolerance independently of each other.

### PrimPol is dispensable for IgV_λ_ hypermutation

**Figure 3.**
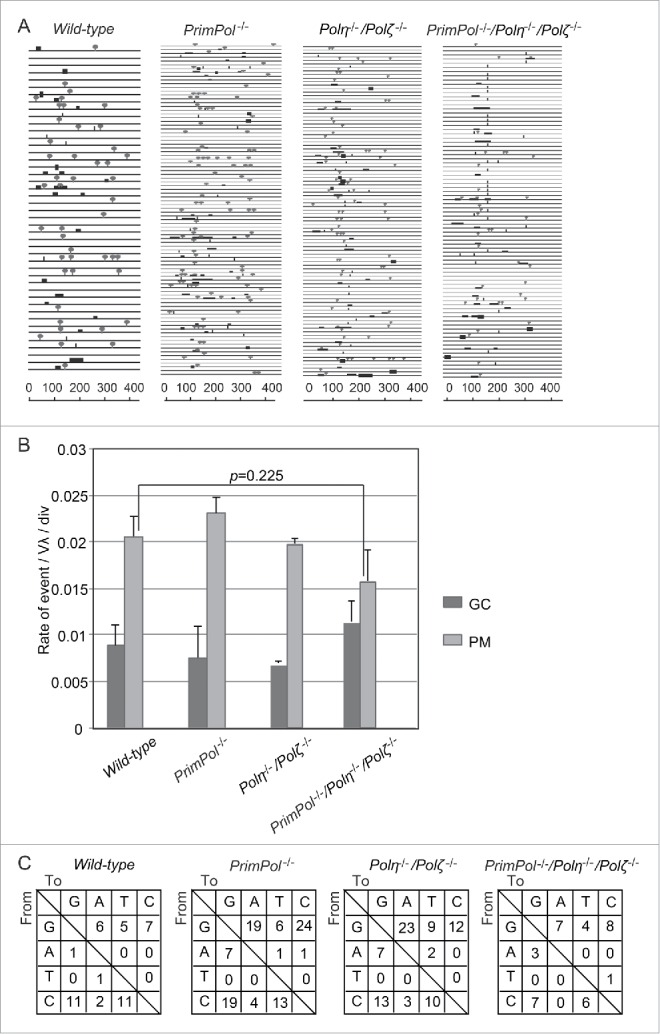
PrimPol is dispensable for TLS past abasic sites during Ig V_λ_ hypermutation. (A) Ig V_λ_ segments isolated from indicated cells, clonally expanded for two weeks. Horizontal lines represent the rearranged Ig V_λ_ (450 bp), with hypermutation (lollipop shapes), gene conversion (horizontal bars), single-nucleotide substitutions that could be the result of hypermutation or gene conversion (vertical bars), and single-base deletion (boxes) determined as described previously.^3,5,29,31,32^ More than three clones were expanded for two weeks and analyzed for each data set. (B) Rate of gene conversion (GC) and hypermutation (PM) events are indicated with standard error. Statistical significance was determined by a Student's *t*-test and *p*-value was calculated. (C) Pattern of point mutation in *wild-type, PrimPol*^−/−^, *Pol*η^−/−^*/Pol*ζ^−/−^ and *PrimPol*^−/−^*/Pol*η^−/−^*/Pol*ζ^−/−^ cells. Tables show the pattern of mutation in each line.

To analyze the roles of PrimPol in TLS *in vivo*, we analyzed the diversification of the IgV_λ_ region, since its non-templated hypermutation at the C/G pair (hereafter called hypermutation) is caused by TLS past abasic sites.^3,5,30-32^ DT40 cells constitutively diversify their IgV_λ_ locus by hypermutation and by gene conversion from pseudo-V_λ_ segments to the downstream VJ_λ_ segment through HR. Therefore, phenotypic analysis of IgV_λ_ diversification during *in vitro* passage provides a novel opportunity to functionally analyze the two alternative mechanisms of releasing replication blockage: TLS and HR^33^ (Fig. S2). Indeed, the rate of TLS dependent IgV_λ_ hypermutation was critically reduced in TLS defective *PCNA*^*K164R*^, *RAD18*^−/−^*, POLD3*^−/−^ and *Pol*η^−/−^*/Pol*ν^−/−^*/Pol*θ^−/−^ cells.^5,3,29^ Notably, the rates of hypermutation and gene-conversion events were similar between *PrimPol*^−/−^ and *wild-type* cells (Fig. 3A-B). Moreover, the mutation spectrum was not significantly changed by the loss of *PrimPol* in *wild-type* and *Pol*η^−/−^*/Pol*ζ^−/−^ cells (Fig. 3C). Notably, *PrimPol*^−/−^*/Pol*η^−/−^*/Pol*ζ^−/−^ cells exhibited only slightly reduced hypermutation levels, demonstrating that TLS is active in the triple mutant (Fig. 3). Taken together, these results indicate that PrimPol is dispensable for TLS dependent IgV_λ_ hypermutation. Considering the serious proliferation defect, as well as the increased sensitivity to damage in the triple mutant, PrimPol might play a critical role in damage tolerance independently of TLS and this role compensates for the TLS defects in *Pol*η^−/−^*/Pol*ζ^−/−^ cells.

### PrimPol's repriming activity is required to tolerate replication-stalling lesions

**Figure 4.**
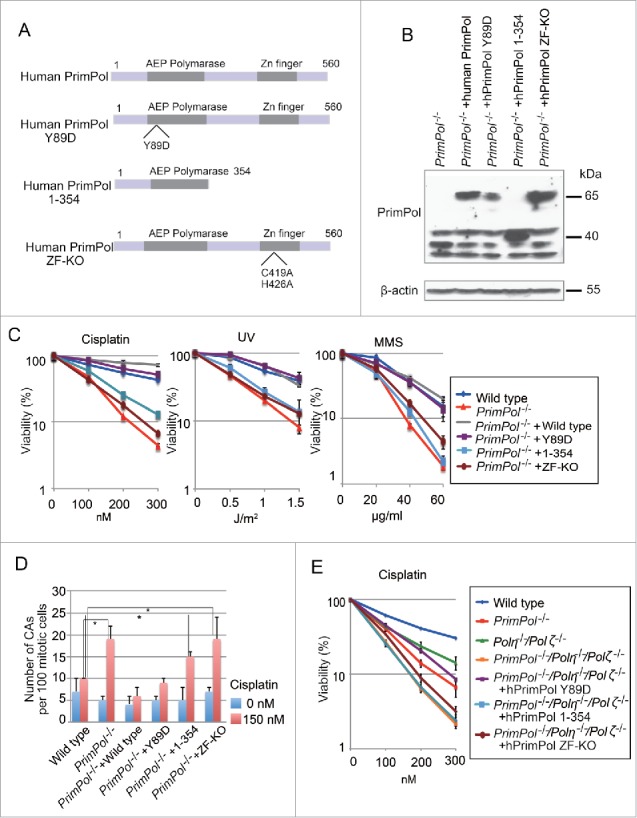
PrimPol's primase activity is required for replication block tolerance. (A) Domain architecture of PrimPol. PrimPol is composed of an AEP polymerase and a Zn^2+^ finger domain. The PrimPol-Y89D mutation is located in the AEP polymerase. The PrimPol1-354 deletion mutant contains the polymerase domain but lacks the Zn^2+^ finger domain. A Zn^2+^ finger domain knockout mutant was generated by mutating the first conserved cysteine and histidine residues that coordinate Zn^2+^ (C419A and H426A, respectively). (B) *PrimPol* ^−/−^ cells were complemented with variants of PrimPol and their *in vivo* expression was confirmed by protein gel blot. Asterisks indicate non-specific bands. (C) Cells with the indicated genotype were exposed to the indicated genotoxic agents. The dose of the genotoxic agent is displayed on the x-axis on a linear scale, while the percent fraction of surviving cells is displayed on the y-axis on a logarithmic scale. Error bars show the SD of the mean for three independent assays. (D) Number of the chromosomal aberrations in 100 mitotic cells was presented. DT40 cells were exposed to cisplatin (150 nM) for 14.5 h and colcemid was added 2.5 h before harvest to accumulate a mitotic fraction. Error bars represent SD of the mean for three independent assays. Statistical significance was determined by a Student's *t*-test and *p*-value was calculated. (*) *p* < 0.05 (E) Sensitivity to cisplatin for indicated cells were indicated as in C.

To test whether PrimPol's repriming activity also contributes to tolerance of other replication-stalling lesions, other than UV,^15,20^ we expressed PrimPol^Y89D^ (reduced TLS, primase active), PrimPol^ZF-KO^ (TLS active, primase defective), and PrimPol^1-354^ (TLS active, primase defective) in *PrimPol*^−/−^ cells (Fig. 4A-B). PrimPol^Y89D^ complemented the reduced damage tolerance of *PrimPol*^−/−^ cells, as did *wild-type* PrimPol (Fig. 4C). This result is consistent with our previous observation that PrimPol^Y89D^ complements increased fork arrest in *PrimPol*^−/−^ cells treated with UV light to the same degree as does *wild-type* PrimPol.^23^ In contrast, neither PrimPol^ZF-KO^ nor PrimPol^1-354^ suppressed hypersensitivity to MMS, UV, or cisplatin in *PrimPol*^−/−^ cells, indicating that the repriming activity of PrimPol, rather than its TLS activity, is pivotal for cellular tolerance to these replication stalling lesions (Fig. 4C). Consistently, the number of chromosome aberrations induced by cisplatin was increased in *PrimPol*^−/−^ cells, *PrimPol*^*−/−*^ +*Primpol*^−/−^+PrimPol^1-354^ and *PrimPol^−/−^*+PrimPol^ZF-KO^ cells, but not in *PrimPol^−/−^*+PrimPol^Y89D^ cells (Fig. 4D). Moreover, expression of PrimPol^Y89D^ but not PrimPol^ZF-KO^ or PrimPol^1-354^ rescued hypersensitivity to cisplatin in *PrimPol*^−/−^*/Pol*η^−/−^*/Pol*ζ^−/−^ cells (Fig. 4E), indicating that this suppression is independent of Polη and Polζ. Together, these findings suggest that PrimPol-mediated repriming of replication plays a critical role in cellular tolerance to a variety of replication stalling lesions and serves as a critical alternative pathway to complement for the potential loss of Polη-Polζ mediated TLS.

### PrimPol's primase activity is required for cellular tolerance of chain terminating nucleotide analogs (CTNA)

**Figure 5.**
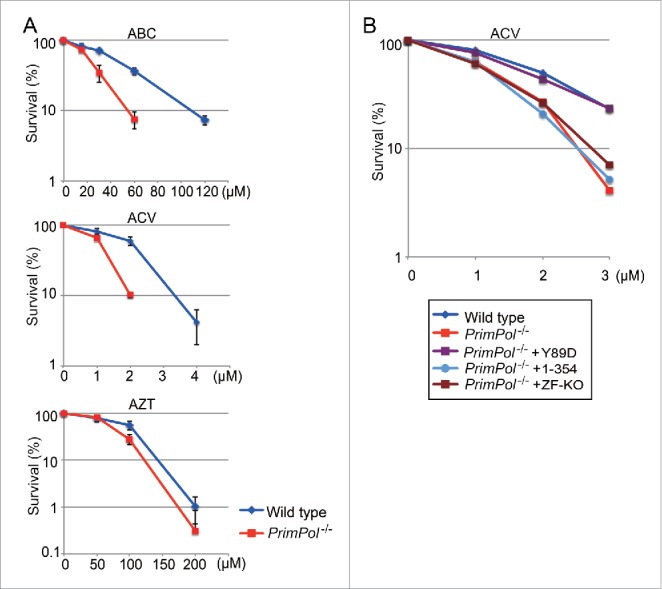
Role of PrimPol's primase activity in cellular tolerance to CTNAs. (A) Cells with the indicated genotype were exposed to the indicated CTNAs. The dose of the genotoxic agent is displayed on the x-axis on a linear scale, while the percent fraction of surviving cells is displayed on the y-axis on a logarithmic scale. Error bars show the SD of the mean for three independent assays. (B) Sensitivity to ACV for indicated cells were indicated as in A.

Given the critical requirement of the primase activity of PrimPol for cellular tolerance to replication stalling lesions, we next analyzed the role of this activity in cellular tolerance to CTNAs. CTNAs cause replicase stalling by preventing polymerases from incorporating further nucleotides when CTNAs are added at the 3′-temini of growing DNA polymers.^34,35^ *PrimPol*^−/−^ cells exhibited more sensitivity to a wide range of CTNAs including Abacavir (ABC),^36-38^ Zidovudine (AZT)^39^ and acyclovir ^40^ than observed with *wild-type* cells (Fig. 5A). Moreover, PrimPol^Y89D^ complemented the reduced CTNA tolerance of *PrimPol*^−/−^ cells, while neither PrimPol^ZF-KO^ nor PrimPol^1-354^ suppressed the increased sensitivity, indicating that the primase activity of PrimPol rather than the TLS activity is pivotal for cellular tolerance to replication stalling induced by incorporation of CTNAs (Fig. 5B).

### PrimPol reprimes replication downstream of CTNA incorporated sites and DNA damage lesions *in vitro*

Given the critical role of PrimPol in the cellular tolerance to CTNAs and the apparent requirement of the enzyme's primase activity for this tolerance, we next tested PrimPol's capacity to reprime downstream of the 3′ side of an incorporated CTNA. Since the ABC drug is phosphorylated in a unique stepwise anabolism to be converted to the triphosphated guanine analog, carbovir, in cells,^41^ we assessed repriming downstream of carbovir, in addition to acyclovir, *in vitro*. In order to analyze repriming downstream of a CTNA incorporation site, a primer containing CTNA (carbovir or acyclovir) at its terminal 3′ end was annealed to a biotinylated DNA template. In addition, we analyzed the ability of PrimPol to reprime downstream of an apurinic/apyrimidinic site (Ap site) and thymine glycol (Tg) lesion located in the template strand, both of which PrimPol is unable to bypass through TLS in the presence of magnesium.^13^ In this case, a 3′ dideoxynucleotide primer was annealed upstream of the templating lesion to represent a situation where replication has stalled at the damage site. The 3′ dideoxy moiety also prevents template-independent primer extension that interferes with the evaluation of PrimPol's repriming activity.

**Figure 6.**
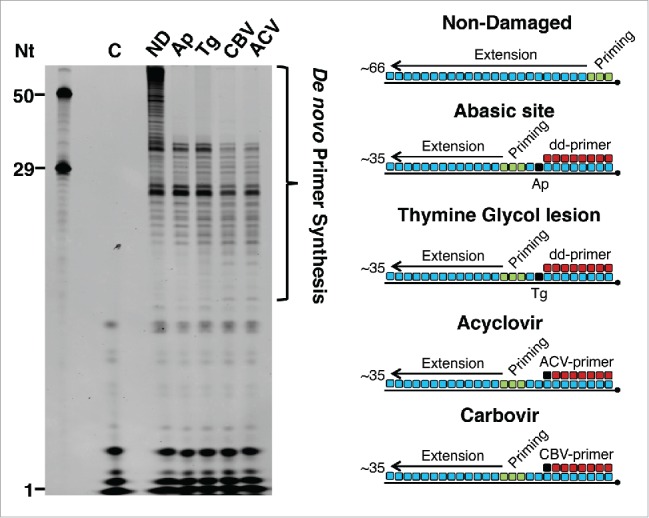
PrimPol catalyzes repriming downstream of 3′ incorporated CTNAs and templating abasic or thymine glycol lesions. PrimPol (1μM) was incubated for 15 min at 37° C with dNTPs (250 μM), FAM-dNTPs (dATP, dCTP, dUTP) (2.5 μM), and mixed sequence primer-templates (1 μM) (as shown in the schematic). Primers containing a 3′ dideoxynucleotide were annealed upstream of the lesion on templates containing a single Ap site (Ap) or thymine glycol (Tg) to allow us to assay for repriming, rather than TLS, activity. In the case of CTNAs, a single CTNA (acyclovir (ACV) or carbovir (CBV)) was located at the 3′ end of the primer in place of the dideoxnucleotide. The length of primase reaction products extended to the end of the template allows analysis of the priming location by PrimPol; the near identical extension products produced in each case show close-coupled repriming by PrimPol downstream of the lesion or CTNA. Oligonucleotide nucleotide (Nt) length markers are shown in the left panel. Priming and extension are represented in the schematic as green and blue, respectively. “C” indicates the no enzyme control. “ND” indicates the non-damaged template without an annealed primer.

Although PrimPol was unable to synthesize through the lesions or extend from 3′ terminal CTNAs, the enzyme displayed a capacity to perform close-coupled *de novo* synthesis of primer strands downstream in each case (Fig. 6). The size of the extended products, both with 3′ carbovir and 3′ acyclovir primers, in addition to the templating Ap site and Tg lesion, were consistent with repriming ∼14 nt downstream of the CTNAs or lesion site. Importantly, in the absence of the CTNA primer or lesion, PrimPol generated longer and more variable synthesis products, indicating that PrimPol is performing close-coupled repriming downstream of a stalled replication fork. Taken together, these results indicate that repriming by PrimPol downstream of an incorporated CTNA or damage site is a potentially important mechanism for maintaining replication in the presence of these potentially lethal chain terminators and DNA lesions.

### Discussion

In this study, we provide further evidence that PrimPol's primase activity, rather than its TLS activity, is required for cellular tolerance to a variety of replication blocking lesions. We also show that although PrimPol is required to maintain replication fork progression at sites of UV-damage,^13,15,16^ we could not detect any gross defect in IgV_λ_ mutagenesis, which is dependent on TLS bypass of abasic sites. Since abasic sites are the most common spontaneously arising lesions in chromosomal DNA,^42^ it is possible that cells have evolved multiple polymerases to continue replication beyond these abundant DNA lesions and any effects due to loss of the PrimPol in IgV_λ_ mutagenesis were masked. Another possibility is that PrimPol facilitates continuous replication beyond lesions via *de novo* repriming of replication downstream from the damage. In accordance with this hypothesis, expression of mutant PrimPol enzyme (PrimPol^Y89D^) possessing reduced TLS activity facilitates the restart of forks arrested at UV lesions in *PrimPol*^−/−^ cells, while mutant PrimPol enzymes (PrimPol^ZF-KO^ or PrimPol^1-354^) defective in primase activity do not correct the fork-progression defect observed in UV-treated *PrimPol*^−/−^ cells.^13,23^ We recently reported that close-coupled repriming by PrimPol is required for replicative tolerance of G quadruplexes in verterbrate cells.^19^ Complementary to these findings, we show here that PrimPol displays a capacity to perform close-coupled repriming downstream of other potential replication fork stalling DNA damage lesions *in vitro*. Since PrimPol is able to extend RNA primers with ribonucleotides, even when bypassing 8-oxoG lesions,^43^ it is possible that TLS by PrimPol also assists transcription at damaged templates.

A point mutation in PrimPol found in familial high myopia (Y89D) is located in the conserved AEP domain.^22^ The link between this mutation and high myopia has been a matter of debate.^44,45^ This mutant enzyme exhibits reduced polymerase processivity.^13,23^ Consistently, cells expressing PrimPol^Y89D^ exhibit reduced fork-progression rates.^13,23^ However, we did not detect any gross defects in chromosome stability or replication-block tolerance (Fig. 4). It is possible that the over-expression of PrimPol^Y89D^ in our assay system masked the effect of reduced polymerase activity in this mutant enzyme. Another possibility is that multiple polymerases, including TLS polymerases and replicative polymerases, compensate for the reduced polymerase activity of PrimPol. Recent studies consistently show a redundancy in the way multiple TLS polymerases are working, and that even replicative polymerase δ compensates for the loss of TLS polymerase.^2,3,5^

How the appropriate damage tolerance polymerase is chosen from among the multiple stalled-fork recovery systems is largely unknown. The similar levels of hypersensitivity to DNA damaging agents found in *PrimPol*^−/−^ and *PrimPol*^−/−^ + PrimPol ^ZF-KO^ cells, in addition to the ability of PrimPol to reprime downstream of DNA damage lesions *in vitro*, has led us to hypothesize that repriming of replication downstream from a DNA lesion might play a more important role in the recovery of stalled forks than previously thought.^46,47^ The simultaneous loss of Polη and Polζ caused no significant decrease in TLS-dependent IgV_λ_ hypermutation but a significant change in the mutation pattern,^2^ suggesting that other polymerases carry out TLS in their absence. Moreover, the loss of PrimPol in *Pol*η^−/−^*/Pol*ζ^−/−^ cells did not significantly further reduce TLS-dependent IgV_λ_ hypermutation. Thus, the contribution of PrimPol to TLS-dependent IgV_λ_ hypermutation, even in the absence of Polη and Polζ, might be limited, if it exists at all. In contrast, *PrimPol*^−/−^ */Pol*η^−/−^*/Pol*ζ^−/−^ cells exhibited critical defects in cellular proliferation and tolerance to DNA damaging agents, suggesting that PrimPol serves as a critical backup for Polη and Polζ in stalled-fork recovery. Thus, PrimPol-dependent repriming of replication might provide a complementary mechanism to facilitate fork recovery by TLS. How repriming of DNA replication is regulated is therefore an important issue to be investigated in future studies.

In this study, we also identified a critical role for repriming by PrimPol in the cellular tolerance of CTNAs. Anti-viral CTNAs can lead to strong mitochondrial toxicity due to their incorporation by the less selective mitochondrial Polγ.^48^ Our findings suggest that repriming by PrimPol might be critical for the completion of DNA replication against CTNAs in both mitochondrial and nuclear replication. The use of chain-terminating nucleoside analogs, in combination with other chemotherapeutic treatments, to kill cancer cells shown to have mutated or low expression levels of PrimPol might be a promising chemotherapy strategy.

## Material and methods

### Disruption of PrimPol in chicken DT40 cells

DT40 cells were provided by the Takeda lab.^27^ PrimPol disruption constructs were generated from genomic PCR products combined with *puro*^*R*^ and *bsr*^*R*^ selection-marker cassettes. Genomic DNA sequences were amplified using primers

5′-CAGTCCAGTAATAAAGAAGGAATCACTTAC−3′ and 5′-CCCATTTTCTCTTCATTTGTCCTAAAGCAA −3′ for the 5′ arm of the targeting construct and 5′- TCAGGAGTCACCGTATCCAGAGATTGATT −3′ and 5′- ATACAGTATTTGGCTTATCAGTAGAAGTTG −3′ for the 3′ arm. PCR was conducted using PrimeStar GXL DNA polymerase (Takara Bio) according to the manufacturer's instructions. An amplified 1.6 kb 3′ arm and a 1.7 kb 5′ arm were cloned into the *Xba*I-*Bam*HI and *Bam*HI -*Sal*I sites, respectively, of a pBlueScript SK vector. Marker-gene cassettes, *puro*^*R*^ and *bsr*^*R*^ selection-marker genes, flanked by loxP sequences, were inserted into the *Bam*HI site to generate *PrimPol-puro*^*R*^ and *PrimPol -bsr*^*R*^. To generate *PrimPol* ^−/−^ cells, *wild-type* DT40 cells were transfected sequentially with *PrimPol-puro*^*R*^ and *PrimPol -bsr*^*R*^. A PCR fragment produced from genomic DNA using primers 5′-ATTCTGCTGAATCAAAACCACCACACAC−3′ and 5′- TCAGCTTCCTGTTTAGATCAGTATGCTC −3′ was used as a probe for Southern blot analysis to screen gene-targeting events.

### RT-PCR

The lack of functional *PrimPol* was confirmed by RT-PCR using primers 5′- CGACAGGCTGATGCTTTCAGATTTGTG−3′ and 5′- CCTTTGGAAGCATCTTCGACTGCTGTG −3′. β-actin transcripts were analyzed using primers 5′-AAAATCAAGATCATTGCCCCACCTGAG−3′ and5′- CCTTCATTCACATCTATCACTGGGGAA−3′ as a positive control for the RT-PCR analysis. PCR was conducted as described earlier.

### Complementation and survival assays in PrimPol^−/−^ DT40 cells

DT40 cells were cultured in RPMI1640 medium (Nakalai Tesque, Kyoto, Japan) supplemented with 10% heat inactivated fetal calf serum (FCS) (Biosera, France, lot No. 10011953), 1% chicken serum (Gibco BRL, Grand Island, HY, USA), 10^−5^ M mercaptoethanol, 50U/ml penicillin and 50μg/ml streptomycin (Nakalai Tesque) at 39.5°. *PrimPol*^−/−^ cells were complemented with transgene as previously described.^13^ Expression of PrimPol in *PrimPol*^−/−^ cells was confirmed by western blot analysis using α-CCDC111 (N-13) antibody (Santa Cruz). We measured the amount of ATP in the cellular lysates to determine the number of surviving cells.^49,50^ Cells were treated with each DNA damaging agent (camptothecin, ICRF193 and HU) in 1 ml of medium using 24-well plates and incubated at 39.5°C for 48 h. To analyze sensitivity to γ-rays, cells were irradiated using a ^60^Co γ-ray source and diluted to 10^4^ cells /ml in 24-well plates and incubated at 39.5°C for 48 h. For UV light, 1 × 10^6^ cells were suspended in 0.5 ml of PBS (phosphate-buffered saline) containing 1% FCS in 6-well plates and irradiated with UVC (254 nm wavelength) and 10 μl of irradiated cells were transfer to 1 ml of medium using 24-well plates and incubated at 39.5° C for 48 h. For MMS, 1 × 10^6^ cells in PBS containing 1% FCS were exposed to MMS for 1 hr at 39.5°C and 10 μl of exposed cells were transferred to 1 ml of medium using 24-well plates and incubated at 39.5°C for 48 h. Then, we transferred 100 μl of medium containing the cells to 96-well plates and measured the amount of ATP using CellTiter-Glo (Promega) according to the manufacturer's instructions. Luminescence was measured by Fluoroskan Ascent FL (Thermo Fisher Scientific Inc., Pittsburgh, PA). Camptothecin and ICRF193 interfere with Topoisomerse I and II, respectively, leading to strand breaks. HU inhibits ribonucleotide reductase and thereby prevents replication. MMS, cisplatin and UV induce chemical modifications in base, leading to replication stall at damaged bases.

### Measurement of sensitivity to CTNAs

To measure the sensitivity to CTNAs, methylcellulose colony survival assays were performed as described previously.^50,51^ ABC (Carbosynth, UK), ACV (Sigma, USA), and AZT (Sigma, USA) were mixed in methylcellulose medium.

### Flow-cytometric analysis of cell cycle progression

To analyze cell-cycle progression, 1 × 10^6^ cells were exposed for 10 min to 20 μM 5-bromo-2′-deoxyuridine (BrdUrd; Nacalai Tesque, Kyoto, Japan), then harvested and fixed with 70% ethanol. Fixed cells were incubated with 2 M HCl containing 0.5% Triton X-100, treated first with mouse anti-BrdUrd monoclonal antibody (BD PharMingen, San Diego, CA), then with FITC-conjugated anti-mouse IgG antibody (Southern Biotechnology Associates, Birmingham, AL). Cells were resuspended in PBS containing 5 μg/ml propidium iodide (PI) for subsequent analysis using FACS Accuri (BD Biosciences).

### AID overexpression by retrovirus infection

*Wild-type, PrimPol* ^−/−^, *Pol*η^−/−^*/Pol*ζ ^−/−^ and *PrimPol*^−/−^*/Pol*η^−/−^*/Pol*ζ ^−/−^ cells were inoculated in 96-well plates to obtain single colonies. Single colonies were picked up and their genomic DNA extracted. After the DNA sequence of the V(D)J locus was analyzed, clones without any mutation or gene conversion in the V(D)J locus were obtained. AID overexpression by retrovirus infection was carried out as described previously.^31,52^ The efficiency of infection was about 70%, as assayed by GFP expression. Ig-gene conversion and hypermutation (events/Ig V segment/div) were calculated at dividing times 6.8, 7.0, 7.5 and 11.6 h for *wild-type, PrimPol* ^−/−^, *Pol*η^−/−^*/Pol*ζ ^−/−^ and *PrimPol*^−/−^*/Pol*η^−/−^*/Pol*ζ ^−/−^ cells, respectively.

### Analysis of Ig V_λ_ diversification

Genomic DNA was extracted at 14 days post-infection. Using primers 5′-CAGGAGCTCGCGGGGCCGTCACTGATTGCCG-3′ at the lead V_λ_ intron position and 5′-GCGCAAGCTTCCCCAGCCTGCCGCCAAGTCCAAG-3′ at the back 3′ site of the JC_λ_ intron, the rearranged V_λ_ segments were PCR amplified, cloned into the plasmid, and subjected to sequence analysis. To minimize PCR-introduced mutations, a high-fidelity polymerase, Prime Star GXL (Takara Bio), was used for amplification. The PCR products were cloned in a TOPO Zero Blunt vector (Invitrogen) and sequenced with the M13 forward (−20) primer. Sequencing analyses were done by Takara Bio. Sequence alignment with DNASIS-MAC v3.3 (HITACHI) allowed identification of changes from the parental sequence in each clone. The classification method used was as described previously.^3,5,29,31,32^

### Chromosomal aberrations

For our chromosomal aberration analysis of the DT40 cells, compound-treated *wild-type* and DNA repair-deficient clones were incubated at 39.5° C for 14.5 h. To arrest cells in the metaphase, 0.06% colcemid (GIBCO-BRL) was added 2.5 h before harvest. Measurement of chromosome aberrations was performed as described previously.^53-56^ Briefly, cells were pelleted by centrifugation, resuspended in 7 ml of 75 mM KCl for 15 min at room temperature, and fixed in 2 ml of a freshly prepared 3:1 mixture of methanol:acetic acid (Carnoy's solution). The pelleted cells were then resuspended in 7 ml of Carnoy's solution and pelleted and resuspended in 1 ml of Carnoy's solution, dropped onto clean glass slides, and air dried. Slides were stained with a 5% HARLECO Giemsa stain solution (Nacalai Tesque) for 20 min, rinsed with water and acetone, and dried. All chromosomes in each mitotic cell were scored at 1000× magnification. Chromosomal aberrations were classified as isochromatid or chromatid gaps, breaks, and exchanges (fusions included triradial, quadriradial, ring, dicentric, and other) according to the ISCN system.^57^

### DNA repriming assays

*In vitro* repriming assays were performed as described previously^19^ using DNA templates containing a single lesion and 5′ biotin modification annealed to 3′-dideoxynucleotide primers (sequences listed in Table S1). Oligonucloetides containing carbovir or acyclovir were chemically synthesized as described previously.^58^ Briefly, PrimPol (1 μM) was incubated at 37°C for 15 mins in 20 μl reactions volumes containing 10 mM Bis-Tris-Propane-HCl pH 7.0, 10 mM MgCl_2_, 1 mM DTT, 250 μM dNTPs or rNTPs, and 2.5 μM FAM dNTPs (dATP, dCTP, dUTP) (Jena-Biosciences). Reactions were quenched with binding-washing buffer (B-W) buffer (10 mM Tris-HCl pH 7.5, 500 mM NaCl, 10 mM EDTA) and supplemented with ∼20 μl streptavidin coated beads. Following DNA capture and washing with B-W buffer, beads were resuspended in 20 μL stop buffer (95% formamide solution with 0.25% bromophenol blue and xylene cyanol dyes) and heated to 95°C for 5 minutes. Reaction products were resolved on a 15% polyacrylamide/7M urea gel for 90 minutes at 21 watts and imaged using a FLA-5100 image reader (Fujifilm).

## Supplementary Material

1191711_Supplemental_Material.pdf
