# Supplementary material for: Repriming by PrimPol is critical for DNA replication restart downstream of lesions and chain-terminating nucleosides
Source: Cell Cycle. 2016 May 26;15(15):1997–2008. doi: 10.1080/15384101.2016.1191711 (PMC4968974; doi:10.1080/15384101.2016.1191711)
Supplement: 1191711_Supplemental_Material.pdf [file kccy-15-15-1191711-s001.pdf]

Supplementary Figure S1

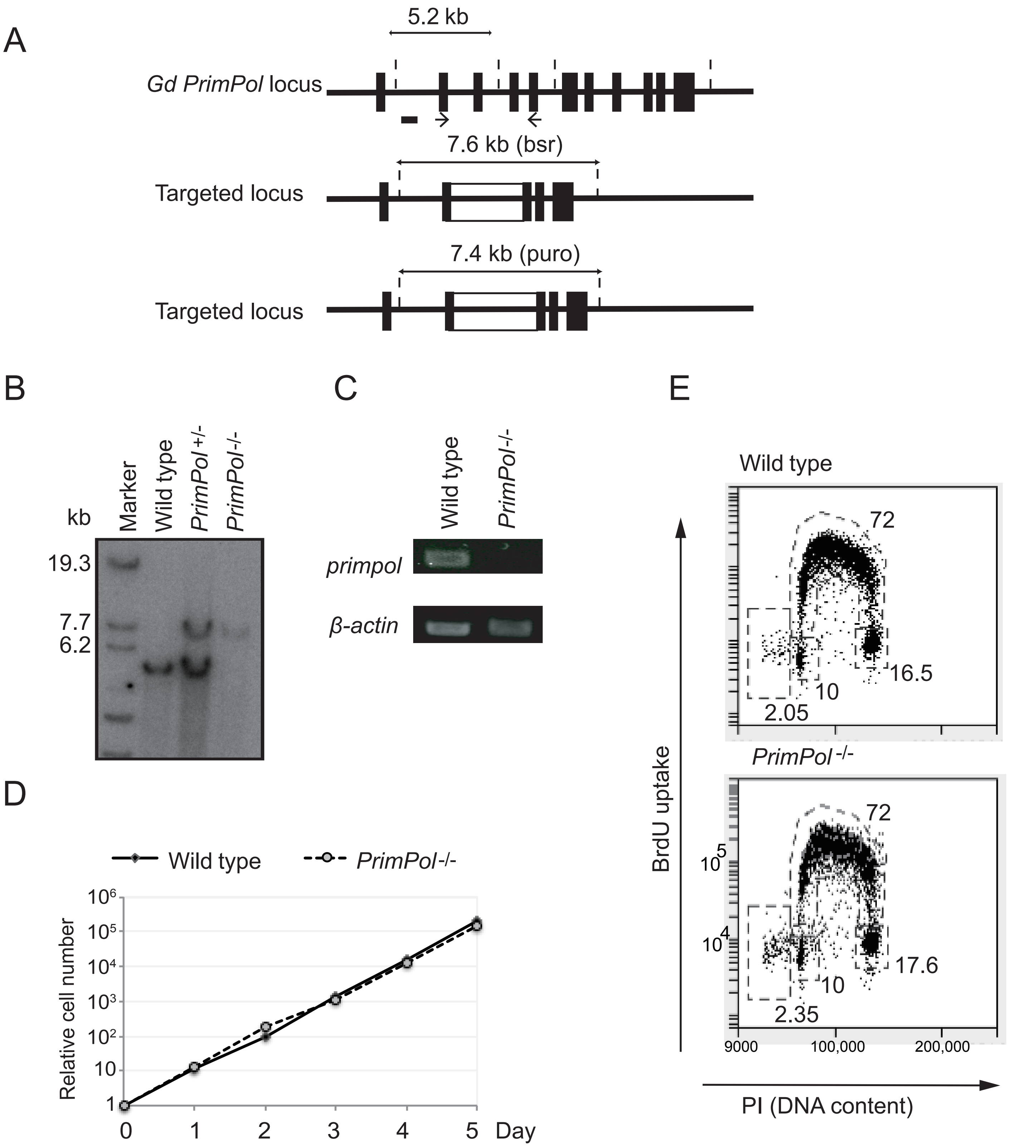

Supplementary Figure S2

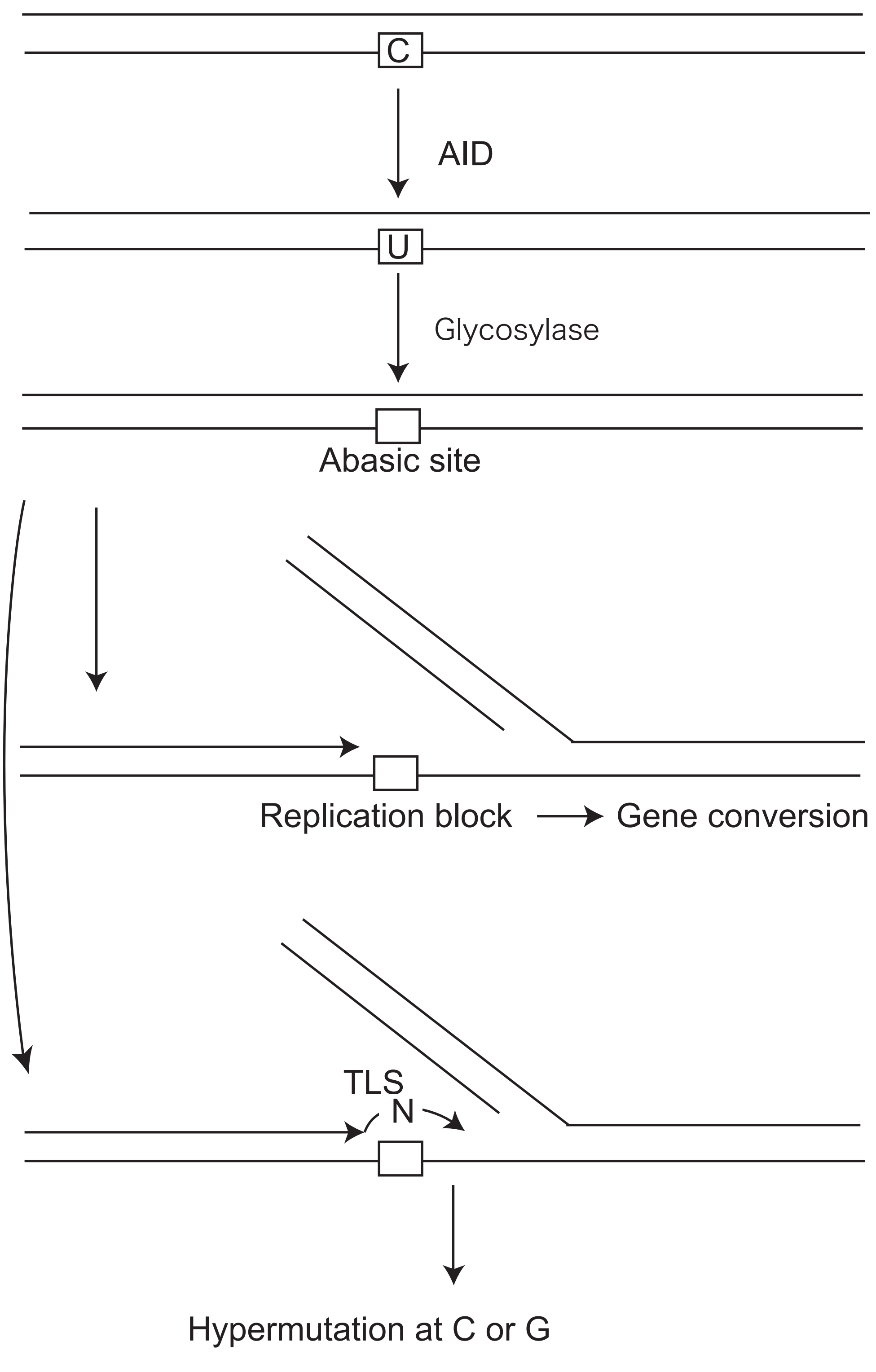

Supplementary figure legends

Figure S1 *PrimPol*$^{-/-}$ cells are able to proliferate with normal kinetics

(A) *PrimPol* disruption in DT40 cells. The wild type chicken *PrimPol* locus from exon 2 to exon 9 was replaced by a *puro* or *bsr* selection-marker gene. Targeted loci (middle and bottom) are shown and compared with the relevant chicken *PrimPol* genomic sequences (top). Solid boxes indicate position of exons. Relevant *Eco*T22I sites and the position of the probe used in the Southern blot analysis are indicated. Arrows indicate the position of the primers used for RT-PCR in (C). (B) Disruption of *PrimPol* confirmed by Southern blot. (C) Depletion of *PrimPol* mRNA in *PrimPol*$^{-/-}$ cells analyzed by RT-PCR (top) using primers designed as shown in (A). β-actin was used as an internal control (bottom). (D) Relative growth rate plotted for the indicated genotypes. (E) Representative cell-cycle distribution for the indicated genotypes. The top, lower left, lower right, and left-most gates correspond to cells in the S, G_1, and G_2/M phases, and the sub-G_1 fraction, respectively. The percentage of cells in each gate is indicated.

Figure S2 AID-dependent cytosine to uracil conversion initiates gene conversion and hypermutation in a chicken IgV_λ segment

AID deaminates cytosine and thereby converts it into uracil. This uracil base is removed by base-excision repair and abasic site is generated. Replication blockage at this site causes gene conversion. Translesion synthesis (TLS) causes hypermutation at the G/C pair.

**Substrate**	**Primer (5'→3')**	**Template (5'→3')**
Non-damaged control	N/A	GTCTTCTATCTCGTCTATATTCTATTGTCTCTATGAATA CCTTCATCAGTCTCACATAGATGCATC
Ap site	GATGCATCTATGTddG	GTCTTCTATCTCGTCTATATTCTATTGTCTCTATGAATA CCTTCATCAApTCTCACATAGATGCATC
Tg lesion	GATGCATCTATGTddG	GTCTTCTATCTCGTCTATATTCTATTGTCTCTATGAATA CCTTCATCATgTCTCACATAGATGCATC
ACV	TCCGTTGAAGCCTGCT TTACV	GTCTTCTATCTCGTCTATATTCTATTGTCTCTATGAATA CCTTCATCCAAAGCAGGCTTCAACGGA
CBV	TCCGTTGAAGCCTGCT TTCBV	GTCTTCTATCTCGTCTATATTCTATTGTCTCTATGAATA CCTTCATCCAAAGCAGGCTTCAACGGA

Supplementary Table 1: Sequences of the DNA oligonucleotides used in the *in vitro* repriming assays. Lesions/modified bases within the sequences are denoted in red.
